# Stem water potential estimation from images using a field noise-robust deep regression-based approach in peach trees

**DOI:** 10.1038/s41598-023-49980-8

**Published:** 2023-12-15

**Authors:** Takayoshi Yamane, Harshana Habaragamuwa, Ryo Sugiura, Taro Takahashi, Hiroko Hayama, Nobuhito Mitani

**Affiliations:** 1 Institute of Fruit Tree and Tea Science, NARO, Tsukuba, 3058605 Japan; 2 Research Center for Agricultural Information Technology, NARO, Tsukuba, 3050856 Japan

**Keywords:** Drought, Plant physiology

## Abstract

Field-grown peach trees are large and have a complex branch structure; therefore, detection of water deficit stress from images is challenging. We obtained large datasets of images of field-grown peach trees with continuous values of stem water potential (Ψstem) through partial secession treatment of the base of branches to change the water status of the branches. The total number of images as frames extracted from videos of branches was 23,181, 6743, and 10,752, in the training, validation, and test datasets, respectively. These datasets enabled us to precisely model water deficit stress using a deep-learning-regression model. The predicted Ψstem of frames belonging to a single branch showed a Gaussian distribution, and the coefficient of determination between the measured and predicted values of Ψstem increased to 0.927 by averaging the predicted values of the frames in each video. This method of averaging the predicted values of frames in each video can automatically eliminate noise and summarize data into the representative value of a tree and is considered to be robust for the diagnosis of water deficit stress in large field-grown peach trees with a complex branch structure.

## Introduction

Advances in deep learning have enabled the adaptation of plant diagnosis methods using digital images in agriculture^1^. For example, the detection of fruits and diseases in plants using images has been shown to be feasible^2–4^. Creating image datasets obtained under actual cultivation conditions is particularly important for the development of image recognition systems^5^. However, the conditions in an open field are complex, and the captured images are affected by noise factors. Sun et al.^3^ stated that changes in the environment and plant status during the day confound the image analysis of plant diseases. There are several factors that are thought to be noisy, such as weather conditions (including sunlight, wind, rainfall, clouds, and time of day), ground conditions (including color and type of soil, amount, and growth of weeds), and structures (including poles, trellis, nets, and plastic sheets), together with the background, including vehicles and workers, among other factors. Particularly in fruit trees, the size of trees is large compared to ordinary crops, the structure of branches is complex, and the growth of branches and light conditions differ greatly between the exterior and interior parts of the same tree. Therefore, image diagnosis of the status of a tree, such as the detection of water deficit stress in an open field, is challenging.

Water deficit stress reduces plant vegetative growth and leaf photosynthesis^6,7^. In contrast, moderate water stress improves the fruit quality by increasing sugar concentration^8^. The effects of irrigation on fruit quality differ depending on the fruit developmental stages^9^. Yield and tree growth are also affected by water management^10^. Therefore, the management of water deficit stress is important for controlling fruit tree growth, yield, and fruit quality in peach trees^11^, and the assessment of plant water deficit stress is invaluable for precise and controlled irrigation.

To assess the water deficit stress, a reliable method for measuring the extent of water stress is of paramount importance. Presently, pressure chamber measurement^12^ serves as a sensitive physiological indicator of tree water deficit stress. The stem water potential (Ψstem), which is measured on a non-transpiring covered leaf in a hermetic aluminum bag, indicates the integrated results of whole-plant transpiration, soil, and root/soil hydraulic conductivity^13^. Ψstem has been used as a stable index of irrigation requirements in deciduous fruit trees^14^, and the relationship between Ψstem and parameters such as vegetative growth, yield and fruit quality, gas exchange, and carbon accumulation has been evaluated in peach and nectarine trees^15–20^.

Although Ψstem is an excellent indicator of water stress of tree, measurement using a pressure chamber has its own drawbacks, such as being destructive and labor-intensive^21^. To counter these drawbacks, nondestructive measurements of Ψstem using other sensors have been developed^22,23^. These measurements can be divided into two categories; intrusive and non-intrusive. Intrusive sensors require intrusive installation into the xylem of the tree, calibration, and maintenance of equipment. Therefore, a fast, low-cost, and non-destructive method is required. Among non-intrusive methods, spectral reflectance indices such as normalized difference vegetation index (NDVI) and the ratio between transformed chlorophyll absorption in reflectance and optimized soil-adjusted vegetation index (TCARI/OSAVI) are known to show correlation with Ψstem; however, these correlations are not enough high (*r*^2^ = 0.68)^24^. These spectral reflectance indices are more closely related to plant vigor than to plant physiological states^25^. Thermal sensing is another non-intrusive method for detecting water deficit stress. However, thermal sensing requires calibration (e.g., wet and dry surfaces) because leaf temperature is affected by both of environmental conditions and stomatal aperture^21^. Our study focused on filling the gap between two spatial scales: the most precise but equally laborious methodologies (pressure chambers) and remote sensing (e.g., unmanned aerial vehicles), which still require improvement with regard to the precision and accuracy of physiological data.

Deep learning has recently been used for digital image-based plant stress phenotyping^1^. Plant images were also used to detect water deficit stress, which was derived from the growth of stem diameter by deep learning in tomato plants^26,27^. The classification of Ψstem from images using deep learning in peach trees has been previously reported^28^. However, these results were obtained for tomato plants or peach trees planted in pots in a greenhouse, and could be applied only to relatively small plants. However, actual peach trees grown in the field are large (e.g., 8 × 6 m in area and 3 m in height) and have a complex structure of main branches, and solar radiation varies between the surface and the interior sections of the tree. Moreover, in natural conditions other unknown factors may contribute to this complexity. Therefore, a different approach of image acquisition and analysis can address the complexities at the field level for the diagnosis of water stress in field-grown trees.

Additionally, in field conditions, it is difficult to generate a dataset of images in which the water deficit stress covers a wide and continuous range of stress levels because the deep and wide distribution of roots and unexpected rainfall in Japan prevent the continuous drying of trees. Therefore, we artificially induced water deficit stress by partial secession treatment of a branch to create a large dataset for learning.

In this study, water deficit stress estimation from images of a peach tree using a field-noise-robust deep regression-based approach is proposed. Our objectives were: 1. Generate a large water deficit stress dataset that could be used to determine the relationship between water deficit stress and image data, 2. Train a deep learning model to assess its effectiveness in water deficit stress modeling using the abovementioned dataset, and 3. Develop a new approach to diagnose water deficit stress in a large tree from extracted frames of a close-shot video of a tree.

## Materials and methods

### Plant materials and water stress treatment

**Figure 1:**
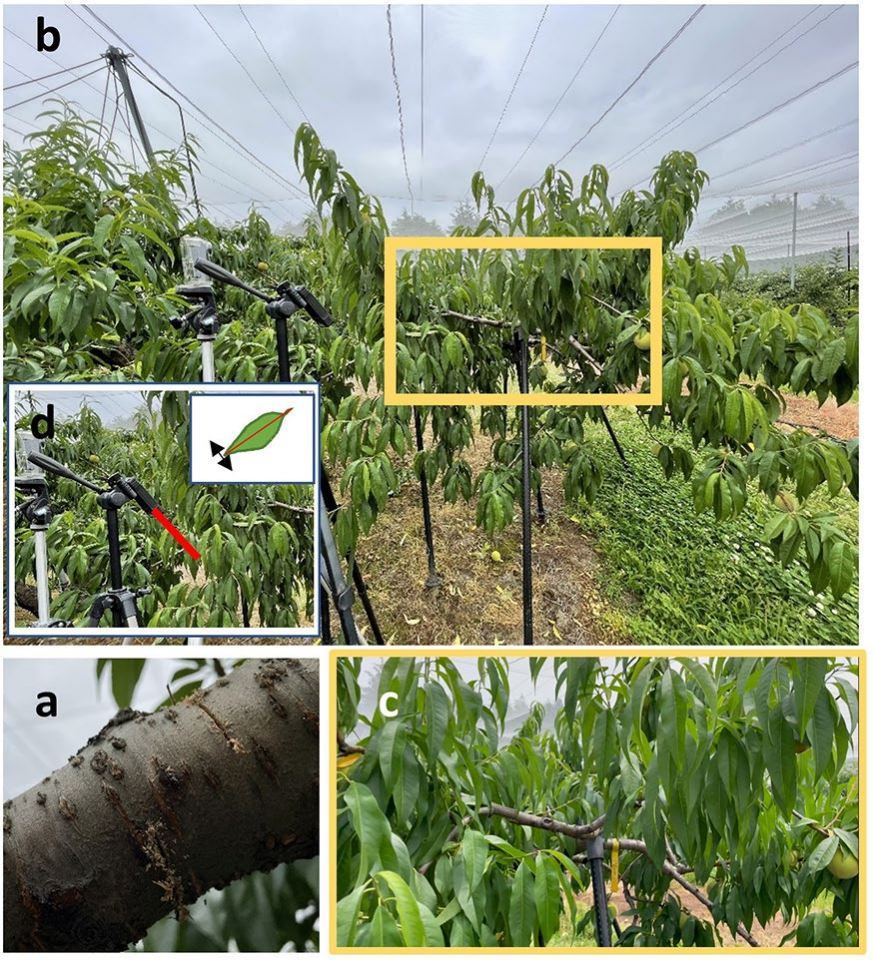
Tree during water deficit stress treatment. (**a**) a partial secession treatment at the bottom of a branch (treatment was conducted from two sides of the branch by using a saw); (**b**) the branch where a video was taken; (**c**) an analyzed frame which was extracted from video; (**d**) the measurement of leaf tip movement by a laser rangefinder.

Five 12- and 13-year-old peach trees (*Prunus persica* (L.) Batsch cv. ‘Akatsuki’) at the National Institute of Fruit Tree and Tea Science, National Agriculture and Food Research Organization (NARO) in Tsukuba, Ibaraki, Japan, were used for the study. The trees were trained to an open vase (8 × 6 m) and conventional commercial production practices were implemented. The orchard was covered with nets to protect from birds, insects and so on. The experiment was conducted from June 8 to 22, 2022. Full bloom occurred on April 5, and the growth stage of these trees ranged from 64 to 78 days after full bloom (stage II of the fruit development stage). Nine secondary scaffolds and lateral branches with lengths ranging from approximately 1–3 m from five trees were used for the partial secession treatment of the base part of the branch using a saw to change Ψstem of the branches to obtain a large dataset for machine learning (Fig. 1a). Partial secession was conducted from the two sides of the branches until the depth of each secession reached just before half the branch diameter. When Ψstem did not change by the treatment, an additional and deeper secession was applied gradually. The timing of the commencement of treatment varied among the nine branches; this step was implemented to obtain a combination of images from various days (for a robust training dataset).

Ψstem was measured on two mature leaves per branch at each measurement time, and two leaves were enclosed in a hermetic aluminum bag for at least 30 min before the measurement. These leaves reached an equilibrium with the main stem of the treated branch; therefore, their water potential represented that of the entire branch. This procedure is known to be a stable method to estimate the water condition^14,29^. Continuous Ψstem values of each branch, which ranged from no stress (− 0.5 MPa) to very high stress (− 2.8 MPa), were obtained on different days and at different times. A pressure chamber (Model 600, PMS Instrument Company, USA) was used to measure water potential.

Videos were recorded around the branches at a distance of 50–100 cm using a smartphone equipped with a camera (iPhone 12 Pro, Apple, Cupertino, CA, USA) when the measurement of Ψstem was conducted (Fig. 1b, c). The image size, video frame rate, and focal length were 3840 × 2160 pixels, 30 frames Per Second (FPS), and 26 mm, respectively. The length of the video depended on the size of the branches and was approximately 30 to 120 s for each time measurement. A total of 107 videos were obtained from nine branches.

The relationship between Ψstem and leaf wilting was confirmed using a laser rangefinder (DISTO X4, Leica Geosystems, Switzerland). A laser rangefinder was set on a tripod, and the distance from the tip of the leaf was measured during the water deficit stress treatment (Fig. 1d).

### Deep learning analysis

A plethora of machine-learning methods can be used for the regression analysis. In recent literature, artificial neural network (ANN)-based deep learning models have shown promising results^30^ compared with other methods. We decided to adopt the commonly available deep-learning models for our purposes. Four pre-trained models, VGG 19^31^, Resnet 50^32^ and 101, and Inception V3^33^ were used for learning. The final linear layer was converted to output a single value, which was used to predict the Ψstem of the branch. The mean square error (MSE) was used as the loss function to fit a regression line when training the models. The datasets of the branches were separated into training, validation, and testing datasets, as listed in Table 1. Independent branches were used for each dataset to avoid overfitting caused by branch type and location. The PyTorch^34^ deep learning framework and Python language were used for training, testing, and validation of the models.

**Table 1:** Changes in Ψstem (MPa) of nine branches (No. 1–9) at each time of measurement during water deficit stress treatment.

Date	Time	No.1	No.2	No.3	No.4	No.5	No.6	No.7	No.8	No.9
8-Jun	9:00					− 0.585	− 0.620	− 0.595	− 0.550	
	11:00					− 2.250	− 2.285	− 0.955	− 1.720	
	11:50					− 2.330	− 2.275	− 1.030	− 1.810	
	14:00							− 1.105	− 1.715	
	16:00							− 0.980	− 1.645	
9-Jun	11:00	− 0.840	− 0.655			− 2.425		− 1.035	− 1.470	
	13:00	− 0.725	− 0.580			− 2.265		− 0.810	− 1.260	
10-Jun	16:00					− 2.835		− 0.625	− 1.260	
13-Jun	10:00	− 1.065	− 0.730					− 1.295		
	13:00	− 1.060	− 0.815		− 0.725			− 1.175		− 0.665
	16:00	− 0.815	− 0.695		− 0.775			− 1.755		− 0.690
16-Jun	10:00	− 0.785	− 2.065		− 0.555			− 1.380		− 0.640
	13:00	− 1.600	− 2.185		− 0.900			− 1.585		− 0.930
	15:00	− 1.385	− 2.230		− 0.880			− 1.380		− 1.305
17-Jun	9:40	− 1.420	− 2.430	− 0.785	− 1.125			− 1.455		− 1.345
	12:00	− 1.750		− 0.795	− 1.695			− 1.555		− 1.850
	14:00	− 1.535		− 0.850	− 1.390			− 1.485		− 1.670
	16:00	− 1.510		− 0.850	− 2.175			− 1.275		− 1.265
20-Jun	10:00	− 2.250		− 1.105	− 2.765			− 1.675		− 1.925
	11:30	− 2.275		− 1.300	− 2.480			− 2.880		− 1.625
	14:00	− 1.960		− 1.275				− 2.050		− 1.590
21-Jun	9:00	− 2.345		− 1.215				− 2.230		− 1.655
	11:30	− 2.290		− 1.155						− 2.225
	14:00			− 2.095						− 2.535
	15:30			− 2.720						− 2.140
22-Jun	10:00			− 2.705						− 2.110
	11:00			− 2.560						− 2.175

## Results and discussion

### Obtained dataset of water deficit stress

**Figure 2:**
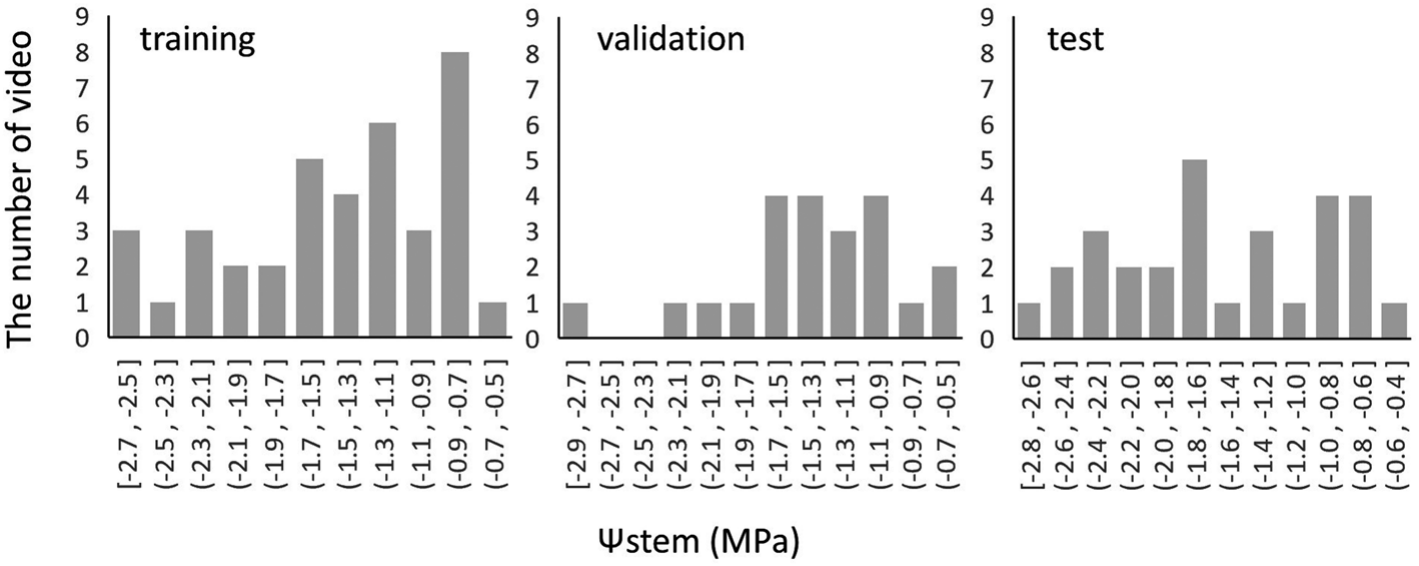
The number of datasets generated and the number of analyzed frames from videos (in parentheses) are 23,181 (31), 6743 (22), and 10,752 (29) in the training, validation, and test datasets, respectively.

The effect of the partial secession treatment on the base of the branch to induce water deficit stress varied among the branches. The actual data for all branches are listed in Table 1. Six branches showed gradual decrease of Ψstem during several days. Three branches (Nos. 2, 5, and 6) showed severe wilting immediately after treatment, and continuous data of Ψstem could not be obtained for these branches. Therefore, six branches were used in this analysis. The six branches were divided into training (Nos. 1, 3, and 8), validation (No. 7), and test (Nos. 4 and 9) datasets to ensure uniform distribution of Ψstem (Table 2, Fig. 2). A total of 82 videos were captured from these six branches (Nos.1, 3, 4, 7, 8, and 9). The average length of the videos was approximately 1 min, and the length of each video varied according to the branch size. Six frames per second were extracted from the video as images, and the total numbers of frames were 23,181, 6743, and 10,752, for the training, validation, and test datasets, respectively.

**Table 2:** Summary of the dataset obtained from nine branches (No. 1–9).

	No.1	No.2	No.3	No.4	No.5	No.6	No.7	No.8	No.9
Ψstem (min)	− 2.345	− 2.430	− 2.720	− 2.765	− 2.835	− 2.285	− 2.880	− 1.810	− 2.535
Ψstem (max)	− 0.725	− 0.580	− 0.785	− 0.555	− 0.585	− 0.620	− 0.595	− 0.550	− 0.640
Number of videos	17	9	13	11	6	3	22	8	18
Usage	Training	Not used	Training	Test	Not used	Not used	Validation	Training	Test

### Learning and analysis of test dataset

**Figure 3:**
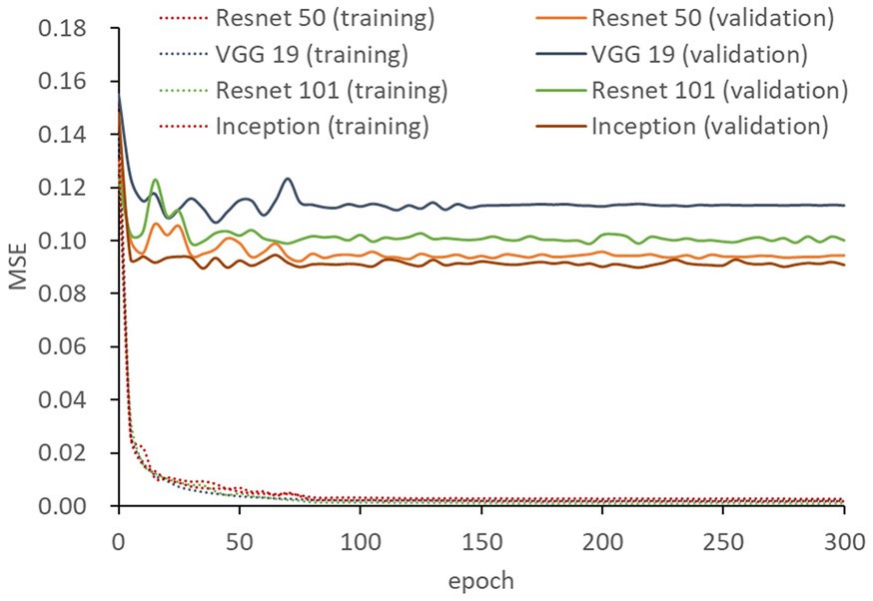
Changes of the mean squared error (MSE) during training by four pretrained models.

**Figure 4:**
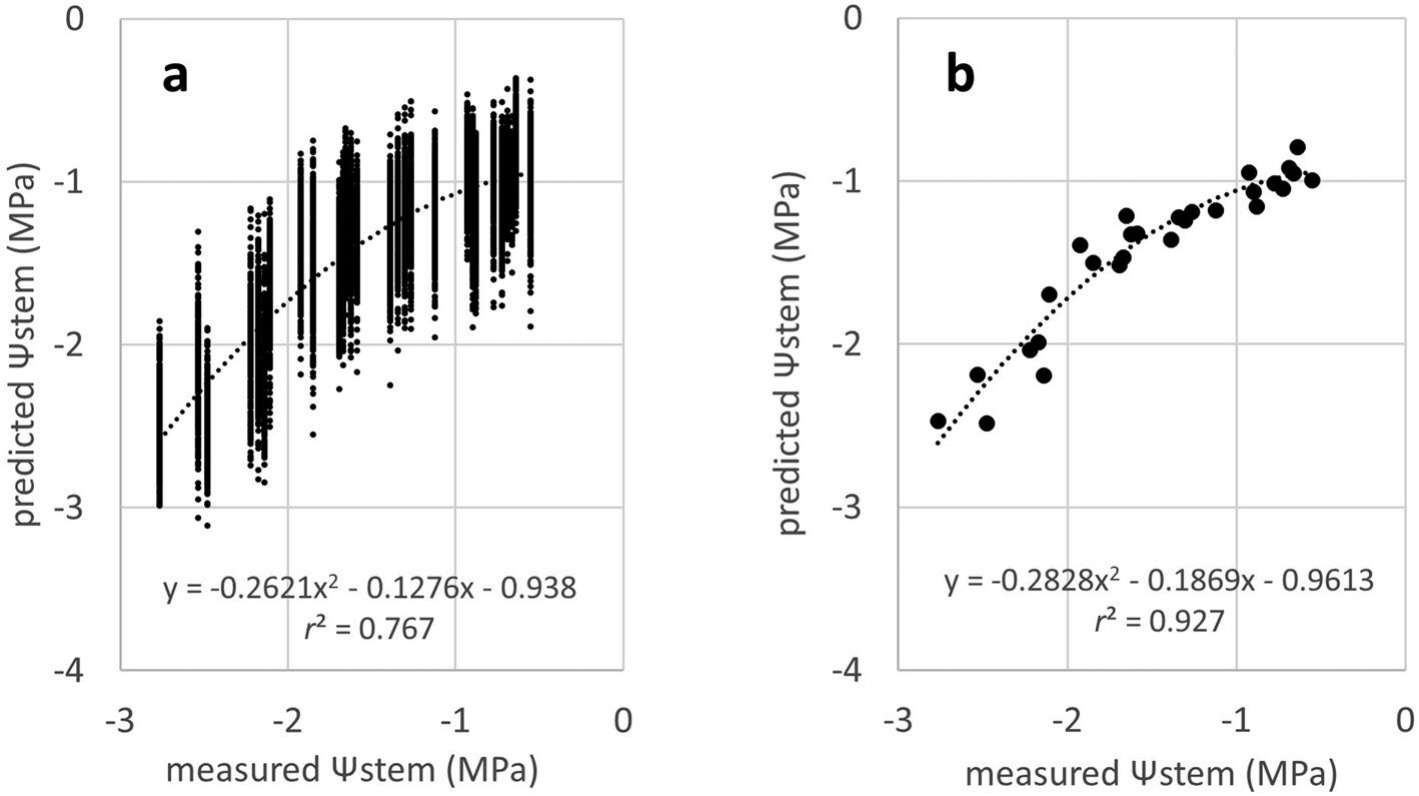
Evaluation of trained model of Reset 50 using the test dataset. (**a**) predicted values of Ψstem of all frames; (**b**) averaged predicted values of Ψstem in each video.

**Figure 5:**
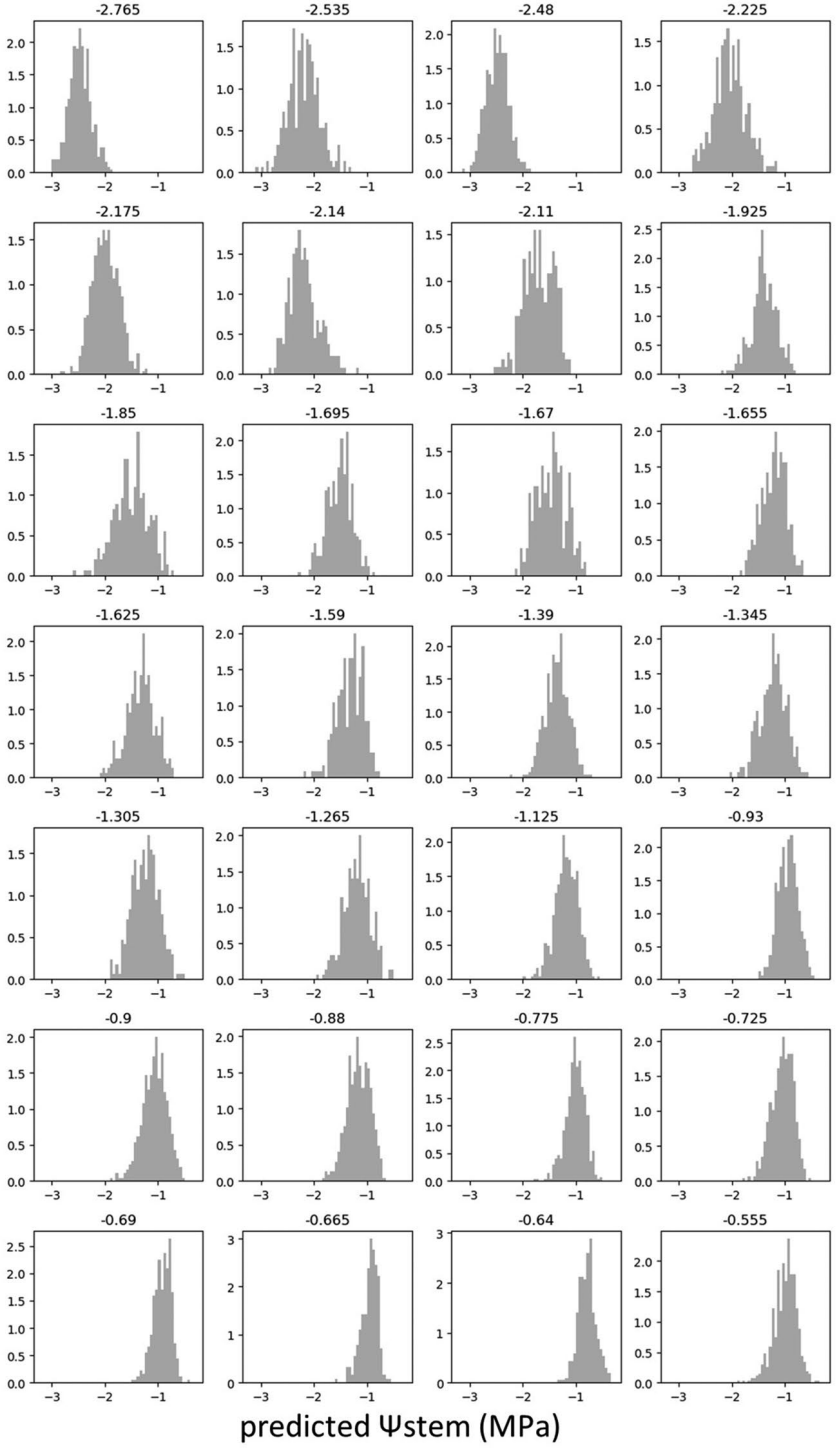
Histogram of predicted Ψstem of frames in each video. The number above each histogram represents the measured Ψstem (MPa) values.

**Figure 6:**
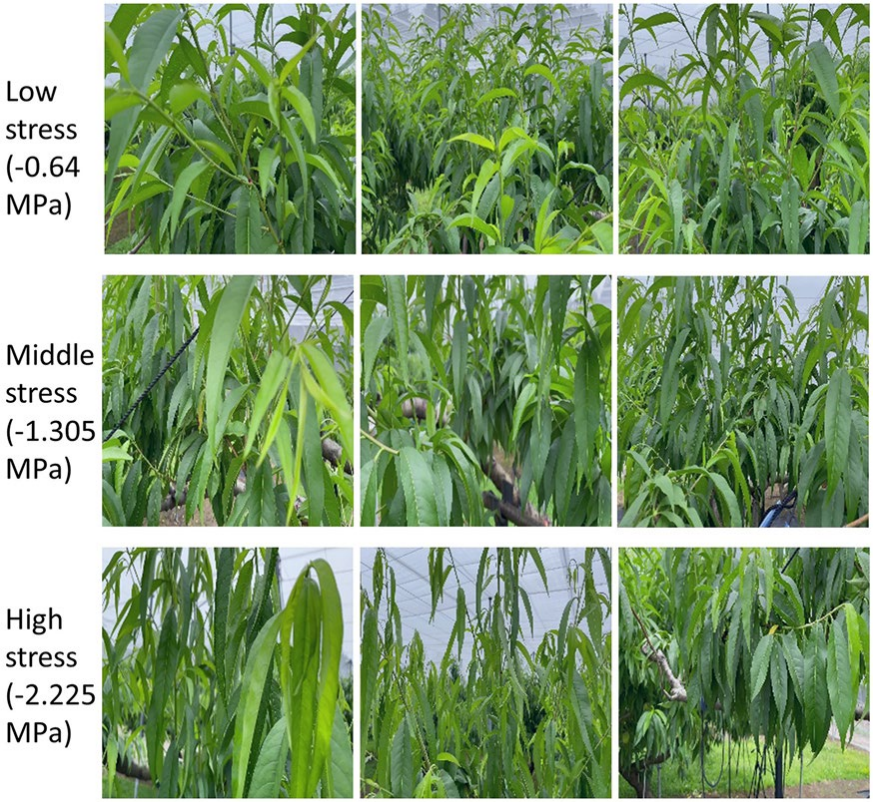
Frames for which predicted values were close to the average values in each video in test dataset. Values shown are the measured Ψstem values.

The MSEs of the training dataset decreased to below 0.003 by 120 epochs in all models (Fig. 3). The lowest MSE (the number of epochs with the lowest MSE) values of validation were 0.1019 (40), 0.0921 (75), 0.0989 (195), and 0.0895 (35), for VGG 19, Resnet 50 and 101, and Inception V3, respectively. Among the four models, Resnet 50 and Inception V3 exhibited a low MSE in the validation dataset. The MSEs in the test dataset, which was evaluated using the trained models of Resnet 50 and Inception V3, were 0.1120 and 0.1167, respectively. As the trained model of Resnet 50 showed a slightly lower MSE than that of Inception V3 in the test dataset, Resnet 50 was used for further analysis. The test dataset was evaluated using the trained model of Resnet 50. The coefficient of determination between measured and predicted values of Ψstem was 0.767 (Fig. 4a). Because the number of frames extracted from each video ranged from approximately 200 to 500, approximately 200–500 predicted values existed for each measured value. Histogram of predicted values of Ψstem for each frame in each video showed a Gaussian distribution (Fig. 5). When the average of the predicted values of the frames in each video was used as the representative value for each prediction, the coefficient of determination between the measured values and the average of the predicted values increased to 0.927 (Fig. 4b). Samples of frames with predicted values close to the average value for each video in the test dataset are shown in Fig. 6.

When the training and test datasets were exchanged (the test dataset was used as the training dataset and vice-versa), the coefficient of determination between the measured and predicted values was 0.7493. The coefficient of determination increased to 0.9005 after averaging the predicted values. Exchanging the dataset did not affect the prediction of Ψstem. When independent branches were used in each of the training, validation, and test datasets, the results suggested that noise factors other than Ψstem such as background, the age and shape of branch, and so on were not learned in the trained model and showed robustness for predicting Ψstem of the trained model.

**Figure 7:**
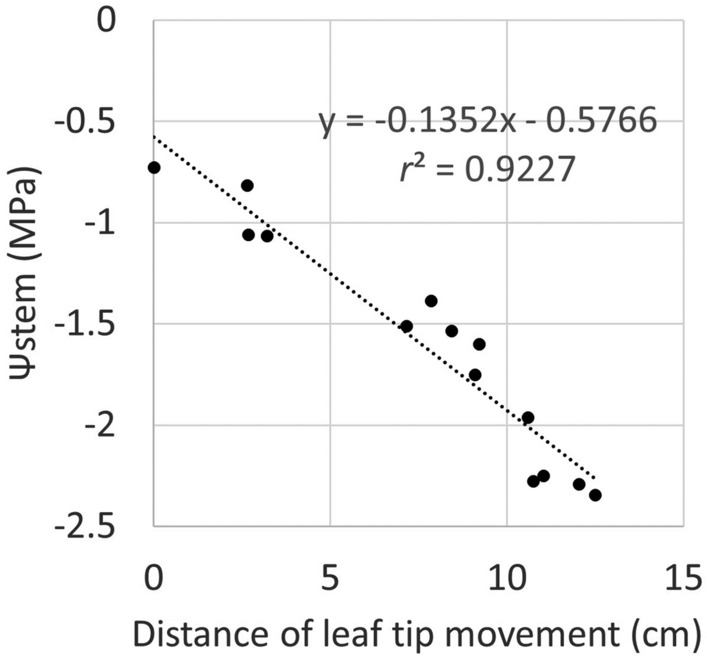
The relationship between distance of leaf tip movement and Ψstem.

Leaf wilting was confirmed by measuring the distance moved by the leaf tip, using a laser rangefinder. Leaf tip movement was highly correlated with Ψstem (*r*^*2*^ = 0.9227), and the relationship was continuous and linear (Fig. 7). This result suggests that changes in leaf shape, including leaf angle, during wilting are strong factors learned by deep learning in the images. The linear relationship between leaf tip movement and Ψstem was confirmed in other trees planted in pots without the partial secession treatment (data not shown). Therefore, changes in leaf shape including leaf angle under water deficit stress condition was not largely different from trees with water deficit stress under natural conditions. Leaf water potential is related to cell turgor^35^. A decrease in cell turgor should occur as the leaves wilt. Engelbrecht et al.^36^ confirmed the relationship between leaf water potential and leaf wilting which was categorized into five classes by visual assessment. Briglia et al.^37^ reported linear relationship between Ψstem and leaf angle which was measured from images of leaves in grape. These results are consistent with those of the present study. In the present study, leaf wilting was measured precisely using a laser rangefinder, and the observed linear relationship between stem water potential and leaf wilting is thought to be useful for interpreting changes in tree appearance during water deficit stress.

Although the partial secession treatment enables the collection of large datasets that are difficult to obtain under natural conditions, a gap between the artificial and natural drying of trees is potentially present. To apply this model to natural trees without secession treatment, a smaller dataset of natural water deficit conditions could be used to adjust the weights of the model by fine-tuning. Continuous efforts to collect a dataset of natural drying conditions, including other growth stages which are one of the noise factors in image diagnosis^3^, are needed to expand the adaptability of the model to different situations.

Regarding noise factors, light conditions, including time of day, weather, and the direction of the camera against the sun, are significant factors affecting the result of the prediction from images using deep learning^3^. In the present study, videos were taken at various time of day with different weather (i.e., sunshine hours during the experiment varied from 0.0 to 11.2 hr per day) from random directions of branches. And the different timing of the partial secession treatment generated different Ψstem of branches at the same time under the same light condition. These methods which actively mix noise factors except for water deficit stress during training were thought to decrease bias and contribute the robust prediction of Ψstem against mainly light conditions. Additionally, the relatively short drying time of the trees by the partial secession treatment is thought to contribute to the elimination of noise induced by changes in the growth stage during the experiment.

### The proposed method of image diagnosis of large trees

Drone images could not be captured because some orchards were covered by nets. We plan to use the camera of a smartphone and/or field robot as future option and focus on plant images captured parallel to the ground. As for the cost estimation when a smartphone is used for diagnosis, the cost of a pressure chamber (e.g., around 3000 to 6000 US dollar in Japan) and the labor of operating it can be reduced, instead of the usage fee of smartphone apps (e.g., 5 US dollars per month).

**Figure 8:**
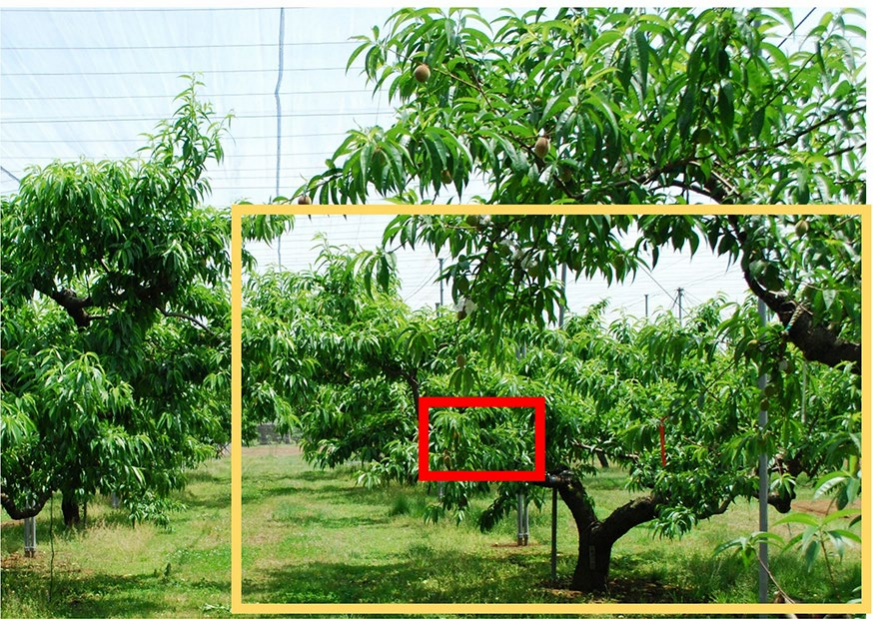
The proposed method of analysis of frames in a close-shot video of a tree; yellow rectangle is a long-shot which covers a whole tree, red rectangle is a close shot of a tree.

Trees grown in the field are large and have a complex structure of main branches, and solar radiation varies between the surface and inside the tree (Fig. 8). Detailed information is lost when capturing an image of an entire tree from a long distance, and the information of an entire tree cannot be obtained when capturing a single image of a small part of a tree from a close distance. Therefore, a different approach for capturing and analyzing images is required to diagnose water stress in field-grown trees. In this study, the extracted frames of close-shot videos of a tree were used for the analysis of deep learning, which can be used to obtain both overall and detailed information simultaneously. Averaging the predicted values of the frames in a video can automatically eliminate noise and summarize the data into the representative value of a tree. Therefore, this method can be useful for image diagnosis of water deficit stress in field-grown trees. Analysis of frames with predicted values near or far from the average can reveal the location where the feature value of the target exists and for the appropriate frames to be taken. Finally, this method can be applied to other image diagnoses of fruit trees grown in the field, such as the nutrient level, vigor, and density of leaves and fruits, because every image diagnosis has the same issues to be addressed. Further research is required to improve and apply this method for the image diagnosis of fruit trees.

### Plant material availability

This study complies with local and national guidelines. Plant experiments were also performed in accordance with the relevant guidelines and regulations.

## Data Availability

All data generated or analyzed during this study are included in this article.
